# Chillblains

**Published:** 1873-01

**Authors:** 


### CHILLBLAINS

ARE caused by a too sudden change from cold to warmth, as in holding a foot almost benumbed with cold too close to the fire; the skin swells and is more or less discolored; they sometimes get partially well of themselves, but often return with the approach of cold weather, accompanied by a very unpleasant itching. As they are caused by cold, the best preventive is to keep the feet always comfortably warm, well protected with thick woolen stockings.

Chillblains are broken or unbroken. If broken, wash the parts, night and morning, with half an ounce of blue vitriol dissolved in half a pint of water; keep it in a bottle and use thus: a teaspoonful night and morning and let it dry in; then when the skin is dry, rub into it with the finger, patiently, any kind of ointment or pain killer, or glycerine or hog's lard; this keeps the skin soft and moist and cool. Meanwhile, avoid approaching the fire suddenly when the affected parts are cold. In moderate cases bathing the parts well in cold water two or three times a day, with patient rubbing in of ointment afterwards, is sufficient.

If the blain is unbroken apply the following ointment, night and morning: melt one ounce each of rosin and beeswax in three ounces of sweet oil, and when thoroughly melted and stirred up, stir in half an ounce of calomine, a preparation of lead which can be had at a drug store.

Another excellent preparation for chillblains and chap is one ounce of collodion, half an ounce of Venice turpentine and two drams of castor oil; apply externally twice a day, and let it dry in.

